# Medial closing wedge distal femoral osteotomy with patient-specific instrumentation: surgical technique, accuracy, and preliminary outcomes

**DOI:** 10.3389/fsurg.2026.1738442

**Published:** 2026-04-15

**Authors:** Leonardo Puddu, Giovanni Lugani, Francesco Perusi, Hansheng Deng, Tianfeng Zhu, Francesco Pisanu, Edoardo Fantinato, Stefano Pescia, Andrea Fabio Manunta, Carlo Doria, Fabrizio Cortese, Gianfilippo Caggiari

**Affiliations:** 1 Department of Orthopaedics and Traumatology, ASUIT Rovereto, Rovereto, Italy; 2 Department of Medicine, Surgery and Pharmacy, University of Sassari, Sassari, Italy; 3 Orthopedic and Traumatology Unit, Azienda Ospedaliero-Universitaria di Sassari, Sassari, Italy; 4 Department of Medicine and Surgery, University of Milano-Bicocca, Monza, Italy; 5 Department of Orthopedics and Traumatology, Mater Olbia Hospital, Olbia, Italy

**Keywords:** closing wedge, knee deformity, malalignment, meniscopathy, osteoarthritis, osteotomy

## Abstract

**Introduction and aim:**

Medial closing wedge distal femoral osteotomy (MCW-DFO) is a surgical technique used to treat symptomatic valgus knee deformity. This retrospective study aims to evaluate the reliability of Patient-Specific Instrumentation (PSI) in reproducing preoperative planning and to assess preliminary clinical and radiographic outcomes in patients treated with MCW-DFO using the PSI technique compared to conventional instrumentation. This research was conducted within the framework of the Italian National Recovery and Resilience Plan (PNRR), Mission 6—Health, as part of the PNRR-MAD-2022-12375978—PEARL Project, supporting the development of precision-based surgical strategies to prevent early osteoarthritis progression.

**Materials and methods:**

Between 2012 and 2023, 34 patients underwent MCW-DFO, of whom 16 were treated with NewClip Technics PSI and met the study's inclusion and exclusion criteria. Preoperative planning was performed using TraumaCad® software, identifying preoperative and planned mechanical femorotibial angle (mFTA) and mechanical lateral distal femoral angle (mLDFA) values. Postoperative measurements were obtained to determine the difference between planned and achieved alignment as an index of surgical reproducibility.

**Results:**

The difference between planned and postoperative values for mFTA and mLDFA angles differed significantly between the two groups. In the PSI group, mean postoperative values differed from planned values by 0.46° for mFTA and 0.66° for mLDFA. In contrast, in the conventional instrumentation group, the difference exceeded 2° for both angles.

**Conclusions:**

The PSI technique proved to be significantly more reliable than traditional instrumentation in adhering to preoperative planning in MCW-DFO. The integration of patient-specific technologies represents a precision-surgery approach consistent with PNRR objectives, potentially improving alignment accuracy and contributing to joint preservation strategies in patients at risk of early osteoarthritis.

## Introduction

Valgus knee deformity is defined as a pathological frontal plane malalignment of the lower limb mechanical axis, characterized by lateral deviation of the weight-bearing line relative to the centre of the knee. This coronal plane malalignment increases the load on the lateral compartment of the knee, accelerating degenerative changes and leading to pain, functional limitations, and reduced quality of life (1, 2). In young, active patients with high functional demands, joint-preserving surgery (JPS) is a viable option that can delay prosthetic replacement and reduce related complications such as revisions and infections (3–5).

The goal of osteotomy is to shift the load axis of the lower limb, relieving the patient's pain, improving joint function, and delaying arthritic progression (6).

A thorough lower limb biometric analysis is essential to determine the origin and extent of valgus deformity, which may arise from the femur, the tibia, or both, thereby guiding the choice of corrective osteotomy (7). To improve the reproducibility of preoperative planning and correction, increasing attention has been given to Patient-Specific Instrumentation (PSI). Surgical options include distal femoral and high tibial osteotomies (medial closing or lateral opening wedge). Among these, distal femoral osteotomy is currently the most frequently used technique for valgus knee correction (8–11).

Distal femoral osteotomy allows direct correction of the femoral-based component of the deformity by addressing lateral femoral condyle hypoplasia at its anatomical origin, which represents one of the most common causes of valgus knee deformity, and can restore joint-line orientation when the deformity predominantly originates from the femur, achieving improved mechanical alignment of the limb without compromising ligament stability (12).

The choice between opening- and closing-wedge osteotomy remains debated, and the available comparative studies have not reached a definitive conclusion (13). However, there is a noticeable trend towards the wider adoption of medial closing wedge osteotomy due to the higher bone consolidation rate, and reduced complications such as irritation of the iliotibial band (14–16).

Osteotomy accuracy and reproducibility of preoperative planning are key determinants of successful distal femoral osteotomy. PSI systems have been developed to translate planning into intraoperative execution and improve alignment accuracy. However, evidence regarding their use in MCW-DFO remains limited.

We hypothesized that the use of patient-specific instrumentation (PSI) in MCW-DFO would improve the accuracy of alignment correction compared with the conventional technique, reduce absolute radiographic errors relative to preoperative planning, and decrease operative and fluoroscopy times without negatively affecting early clinical outcomes.

Therefore, the aim of this study was to (i) describe the MCW-DFO technique using NewClip Technics PSI, and (ii) evaluate its accuracy in reproducing preoperative planning as well as its short-term clinical and radiographic outcomes observed during the early postoperative follow-up period, including functional scores, alignment correction parameters, and radiographic healing assessment, in comparison with a conventional technique.

## Materials and methods

This retrospective case-control study evaluates the accuracy and clinical outcomes of NewClip Technics® PSI compared to the conventional surgical technique of medial closing wedge distal femoral osteotomy (MCW-CFO) for valgus knee correction. A series of consecutive patients diagnosed with knee valgus deformity associated with various degrees of lateral unicompartmental osteoarthritis were treated at the Orthopaedic Department of Santa Maria del Carmine Hospital in Rovereto (TN), from January 1, 2013, to December 31, 2023. They were assigned to two groups based on the surgical technique employed. All surgical procedures were performed by 3 experienced senior orthopaedic surgeons specialized in lower limb deformity correction.

Surgical indications for medial closing wedge distal femoral osteotomy included symptomatic valgus knee deformity in patients younger than 65 years with lateral unicompartmental osteoarthritis (Kellgren–Lawrence grade ≤3), exclusive lateral knee pain, a knee range of motion (ROM) greater than 120° of flexion, and an extension deficit of less than 10°. On weight-bearing full-length radiographs, the deformity was required to be predominantly femoral in origin, with a mechanical femorotibial angle (mFTA) <175° and a mechanical lateral distal femoral angle (mLDFA) <85°. For this retrospective study, patients were included only if they fulfilled all surgical indications and had complete preoperative and postoperative radiographic data available for analysis. Additional inclusion criteria were a minimum radiographic follow-up of 6 months and the availability of standardized full-length weight-bearing radiographs suitable for accurate measurement of mFTA and mLDFA. Exclusion criteria included a clinical history of previous knee intra-articular fracture, knee surgery, or ipsilateral hip arthroplasty. Additionally, patients affected by systemic inflammatory conditions (such as arthropathies, rheumatoid arthritis, or neoplastic pathologies) or fibromyalgia were excluded from the study to limit confounding factors related to pain perception and ROM limitation (Table 1).

**Table 1:** Inclusion and exclusion criteria.

Inclusion criteria	Exclusion criteria
Age ≤ 65 years	Previous surgical interventions
Lateral unicompartmental osteoarthritis (Kellgren Lawrence ≤ 3)	Outcomes of intra-articular knee fractures
Exclusively lateral pain (Finger test +)	Inflammatory arthropathies
ROM in flexion > 120°	Rheumatoid arthritis
Extension deficit < 10°	
Knee valgus > 5° (mFTA < 175°)	Neoplastic pathology
Exclusively femoral valgus (mLDFA < 85°, mMPTA in range)	Fibromyalgia
Clinical and RX follow-up > 12 months	Ipsilateral hip arthroplasty

**Figure 1:**
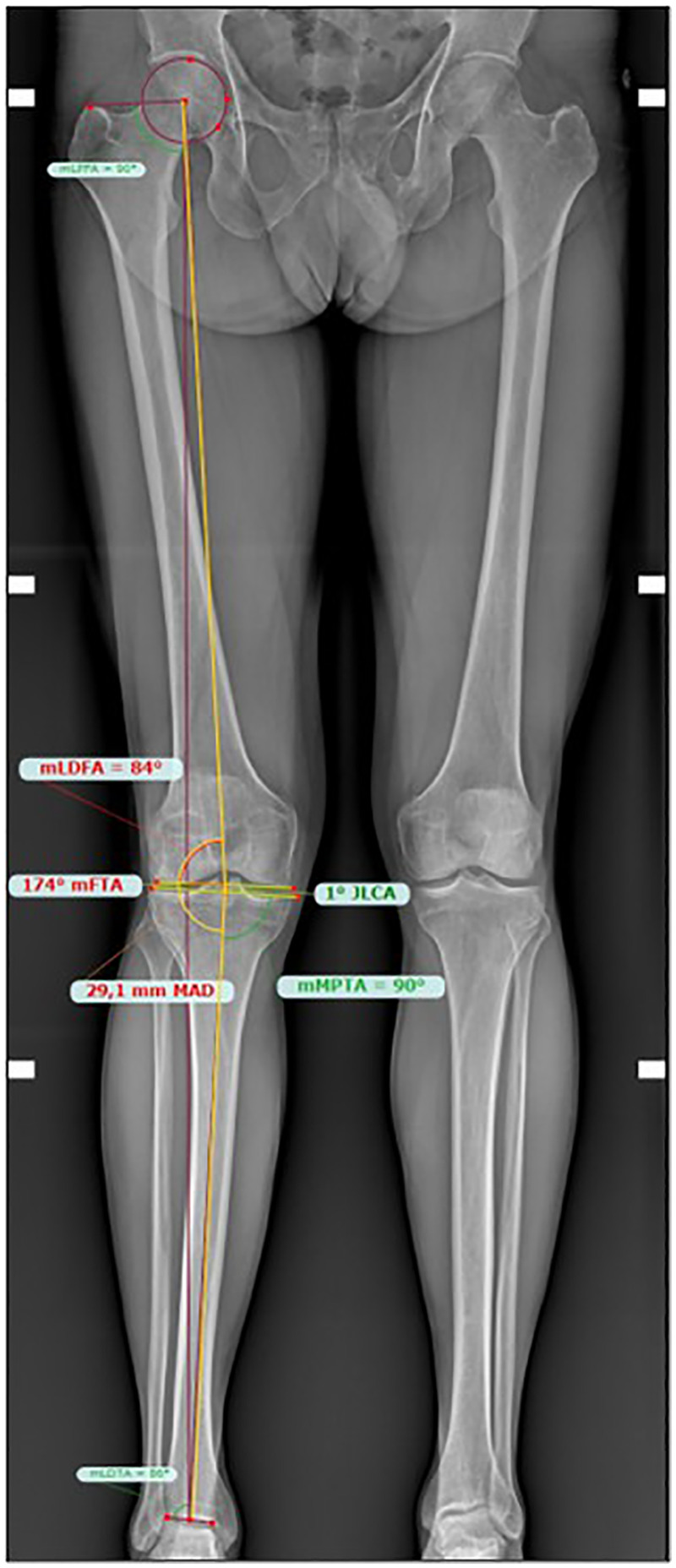
Preoperative biometric evaluation (mFTA, mLDFA, mMPTA, JLCA, MAD).

All patients scheduled for osteotomy routinely underwent a full-length weight-bearing x-ray of the lower limbs, on which preoperative planning was conducted by the surgical team using TraumaCad® software, measuring and tabulating preoperative mFTA and mLDFA values (Figure 1).

**Figure 2:**
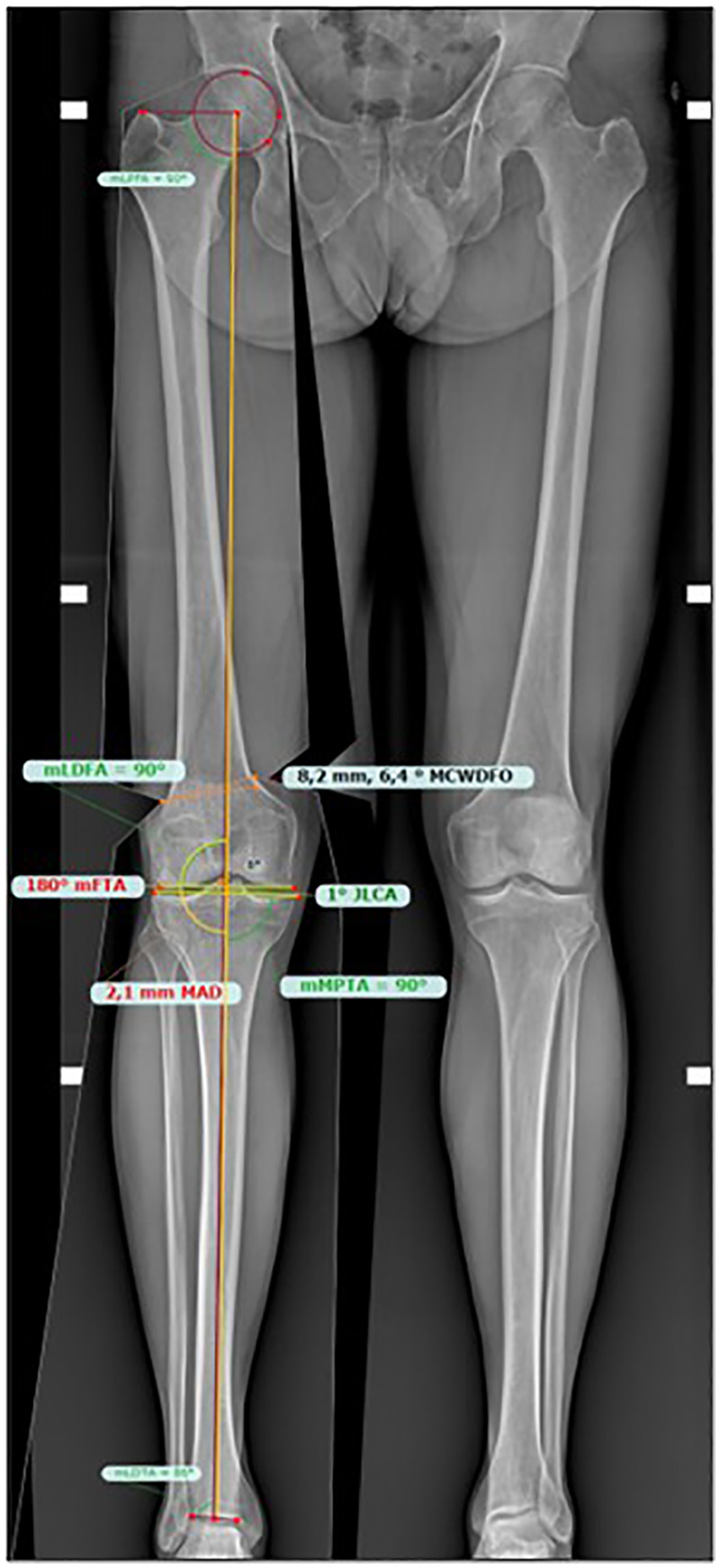
MCW-DFO: preoperative planning.

Patients scheduled for surgery with PSI instrumentation also underwent a preoperative CT scan of the entire lower limbs, with images processed by NewClip Technics® software. Based on the preoperative planning selected by the surgeons, the software facilitated the creation of patient-specific cutting guides for osteotomy. For all patients scheduled for MCW-DFO, planning involved medially translating the load axis of the lower limb so that it intersected the center of the knee, represented by the centre of the tibial spines (Figure 2), and the planned values of mFTA and mLDFA angles were recorded.

**Figure 3:**
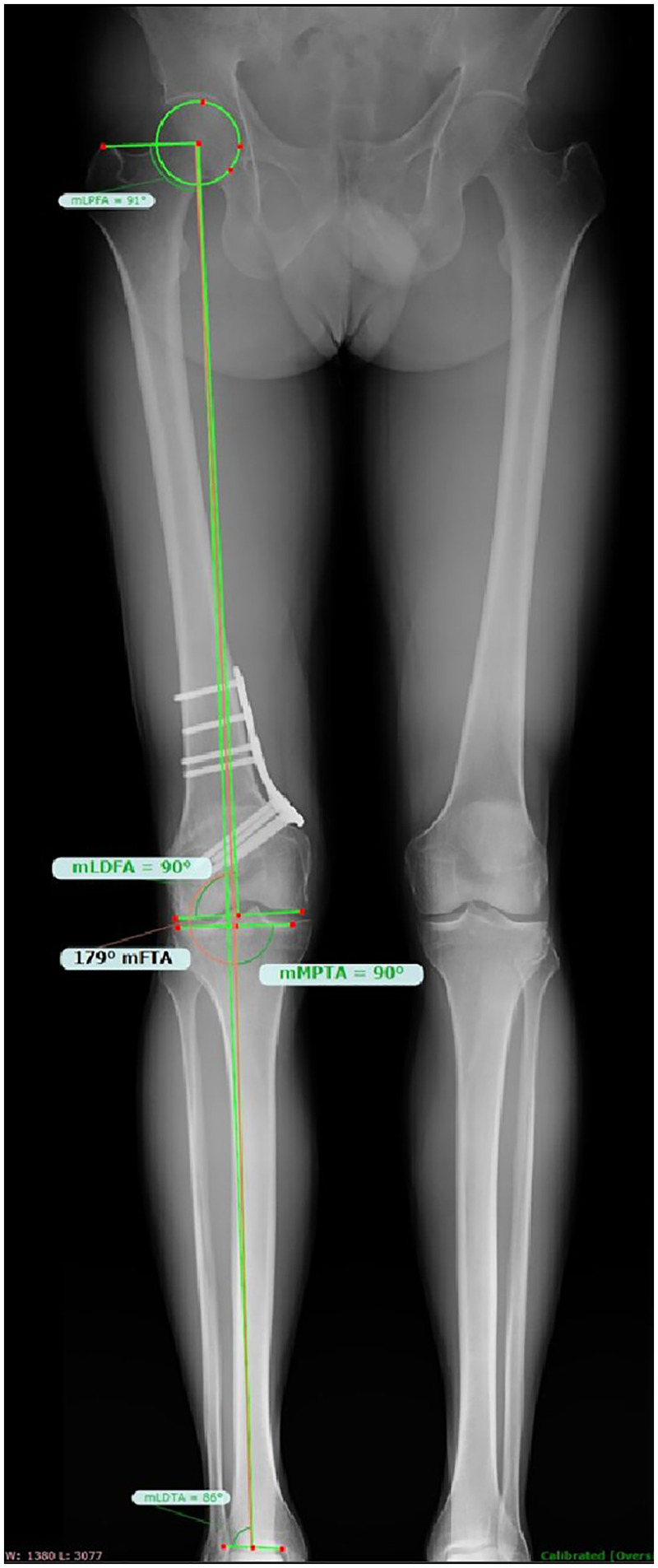
Postoperative Re-evaluation on full-length x-ray.

Each patient in the two observational groups received clinical and radiographic follow-up at 1, 3, 6, and 12 months postoperatively. After 6-month follow-up, a full-length weight-bearing x-ray of the lower limbs was conducted, and postoperative mFTA and mLDFA angles were measured by a single senior surgeon (Figure 3).

Radiological alignment assessment included measurement of the mFTA and the mLDFA on full-length, weight-bearing radiographs. The mFTA was used to evaluate global lower limb alignment, reflecting the overall coronal deviation of the mechanical axis relative to the knee joint (17). The mLDFA was used to assess the segmental contribution of the distal femur to valgus deformity, as it quantifies the angular relationship between the mechanical axis of the femur and the distal femoral joint line (18, 19). These two parameters are widely validated and represent the standard radiographic measures for determining both the magnitude and the anatomical origin of coronal plane malalignment.

Postoperative mFTA and mLDFA values were used to evaluate the corrective effects of the two surgical methods on genu valgum deformity. In addition, the study also evaluated the differences between the two surgical techniques by calculating and comparing the absolute error between the planned and postoperative values, to assess the accuracy and reproducibility of each technique in achieving the intended correction during surgery.

Upon re-examining the 34 patients who underwent surgery, 8 were excluded from the study because they did not meet the inclusion and exclusion criteria. Of the remaining 26 patients, 10 were treated with the conventional technique (MCW-DFO-CONV group), while 16 were treated using PSI (MCW-DFO-PSI group).

### Statistical methods

The primary aim of this study is to assess whether PSI enhances the reproducibility of preoperative surgical planning in MCW-CFO. Absolute error was defined as the absolute value of the difference between the planned and postoperative angles $\left(\varepsilon =|\theta_{\mathrm{planned}}-\theta_{\mathrm{post}\text{-}\mathrm{op}}|\right)$, reflecting the accuracy of surgical execution. This was evaluated by comparing, the planned vs. postoperative values of the mFTA and the mLDFA in each patient across the two groups, providing a quantitative assessment of alignment correction.

Secondary endpoints addressed aspects of surgical efficiency and safety, including operative time and fluoroscopy duration. Additional parameters included preoperative and 12-month postoperative ROM and IKDC scores, to assess the impact of PSI on functional recovery, alongside the occurrence of intraoperative or postoperative complications, allowing for a broader comparison between the two surgical techniques (20).

Data collection, carried out by a single operator, involved selecting candidates through a standardized assessment process that evaluated demographic information, clinical history, minimum average follow-up, as well as pre- and post-operative clinical scores and radiological evaluations.

The statistical analysis of the data included representing categorical variables as counts and percentages and continuous variables as means with standard deviations (SD). Continuous variables were assessed for normality using the Shapiro–Wilk test, and variables conforming to a normal distribution were compared using Student's *t*-test, with Levene's test applied to verify equality of variances. Non-normally distributed variables were analyzed using the Mann–Whitney *U* test. Categorical variables were compared using the Chi-square test or Fisher's exact test, as appropriate. Student's *t*-test and Mann–Whitney test were performed using JASP, JASP Team (2024) (Version 0.19.3) [Windows] to assess significant differences between two groups (*p* < 0.05).

### Surgical technique with PSI instrumentation

**Figure 4:**
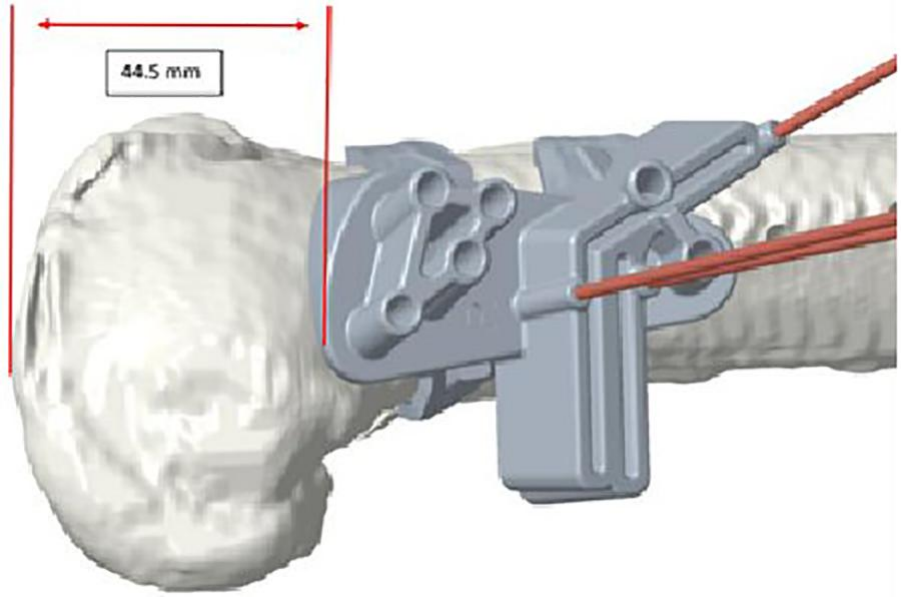
Distance between the cutting guide and the medial joint line.

All procedures were performed by senior consultant orthopaedic surgeons with extensive experience in knee osteotomy procedures. The procedure is performed with the patient in the supine position, using a wedge to maintain the hip and knee flexed at approximately 30°. A pneumatic tourniquet is applied at the root of the thigh, and a sterile field is prepared to cover the entire lower limb. The primary surgeon positions themselves medially to the patient's thigh, while the fluoroscopy device is situated laterally. A straight longitudinal incision is made, starting from the medial femoral epicondyle and extending proximally as necessary, based on the case and the patient's physical constitution. The preoperative planning provided by NewClip Technics® also indicates the distance in millimeters from the joint line where the cutting guide must be placed, offering guidance on the size of the surgical incision (Figure 4).

After passing through the subcutaneous layer and achieving haemostasis, the muscular fascia is identified and incised along the posterior edge of the vastus medialis muscle belly, continuing subvastus to access the femoral diaphysis and metaphyseal region.

**Figure 5:**
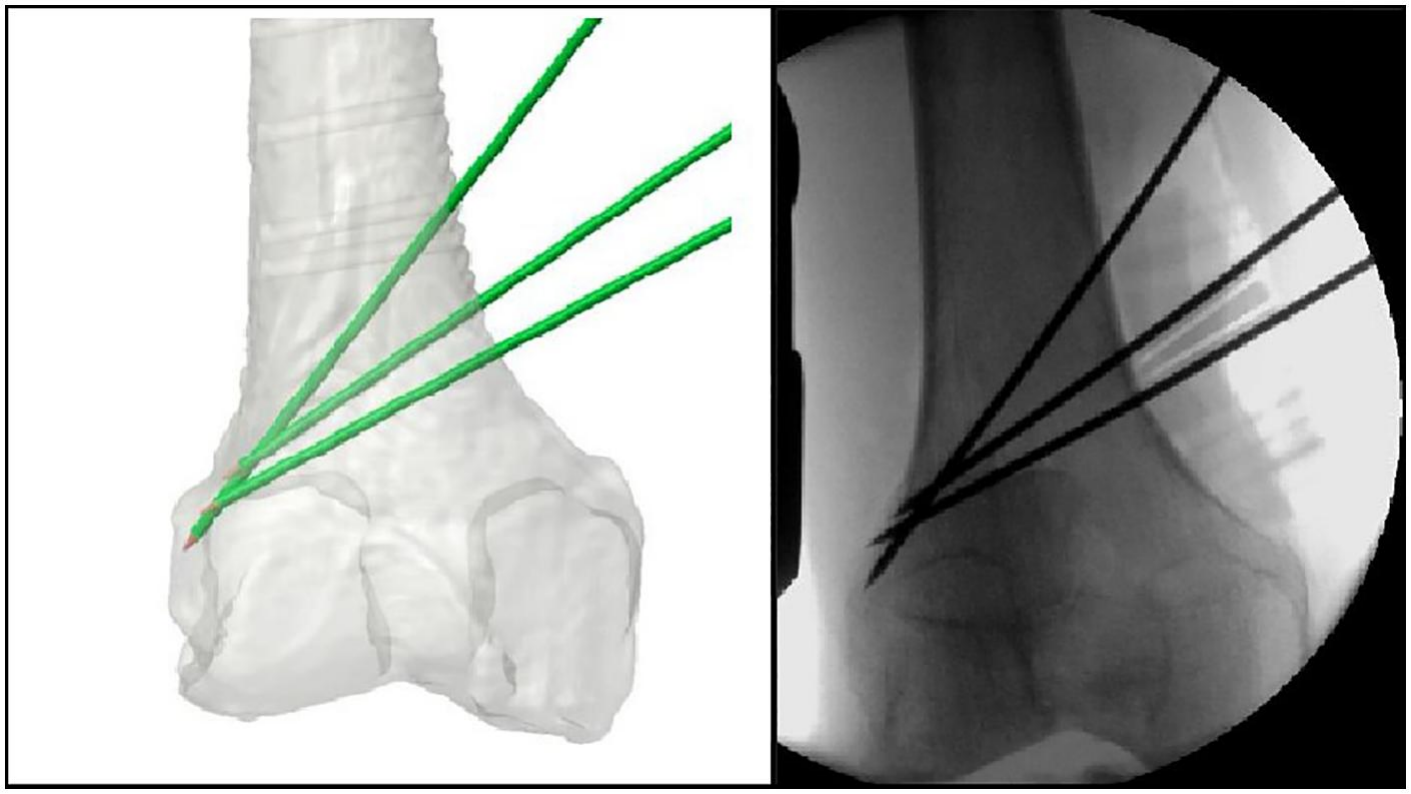
Comparison between the intraoperative positioning of the cutting guide and the position indicated by the preoperative plan.

At this stage, it is essential to correctly position a Homann or Putti retractor behind the femur to safeguard the adductor canal, which houses the neurovascular bundle. After reaching the bone plane, the cutting guide is placed for optimal adherence, which may sometimes necessitate releasing the proximal section of the medial joint capsule by a few millimetres. Because the preoperative simulation is based on CT images that visualize only bony structures, precise intraoperative positioning can be influenced by surrounding soft tissues, cartilage, and periosteum. To mitigate this, careful subvastus dissection is performed to expose the relevant bone surface while preserving soft tissue integrity. The guide is then temporarily secured with at least one 2 mm Kirschner wire from the NewClip Technics® set, utilizing the designated slots on the guide. Intraoperative fluoroscopy in two projections is used to verify alignment relative to the preoperative plan, and minor adjustments are made as needed to ensure the guide conforms as closely as possible to the simulated position. This step is critical to maintain the accuracy of osteotomy and achieve the planned mechanical alignment (Figure 5).

**Figure 6:**
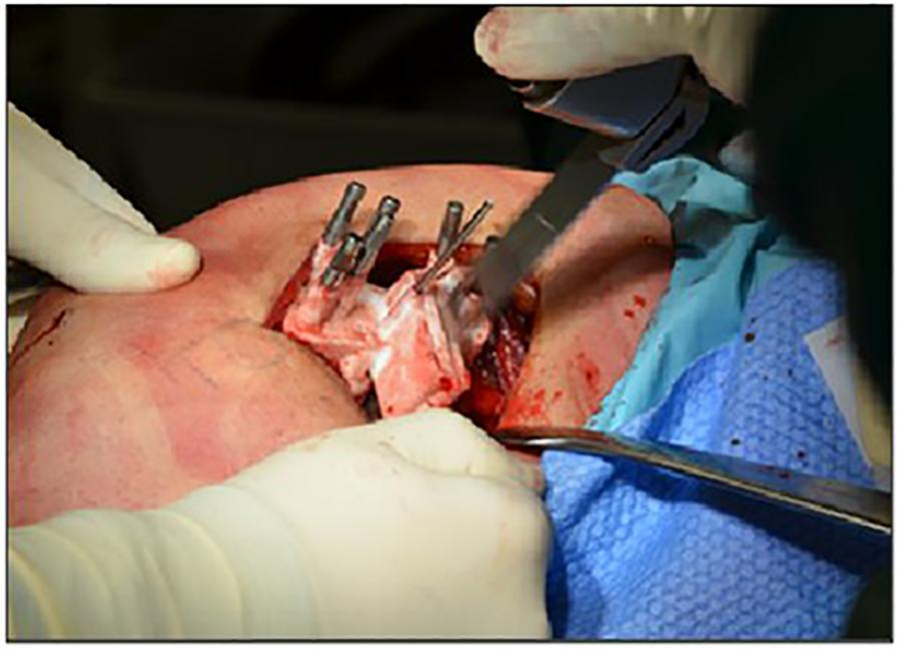
Execution of the osteotomy with the oscillating saw using the slots of the patient-specific cutting guide.

Once the correct position is achieved, a third Kirschner wire is placed to protect the lateral osteotomy hinge, and the guide is further stabilized with pins positioned after drilling the femur at the precise location where the final screws will be inserted. The osteotomy is then performed by sliding the oscillating saw into the designated slots of the patient-specific cutting guide, being careful not to exceed the osteotomy depth indicated by the preoperative plan (Figure 6).

**Figure 7:**
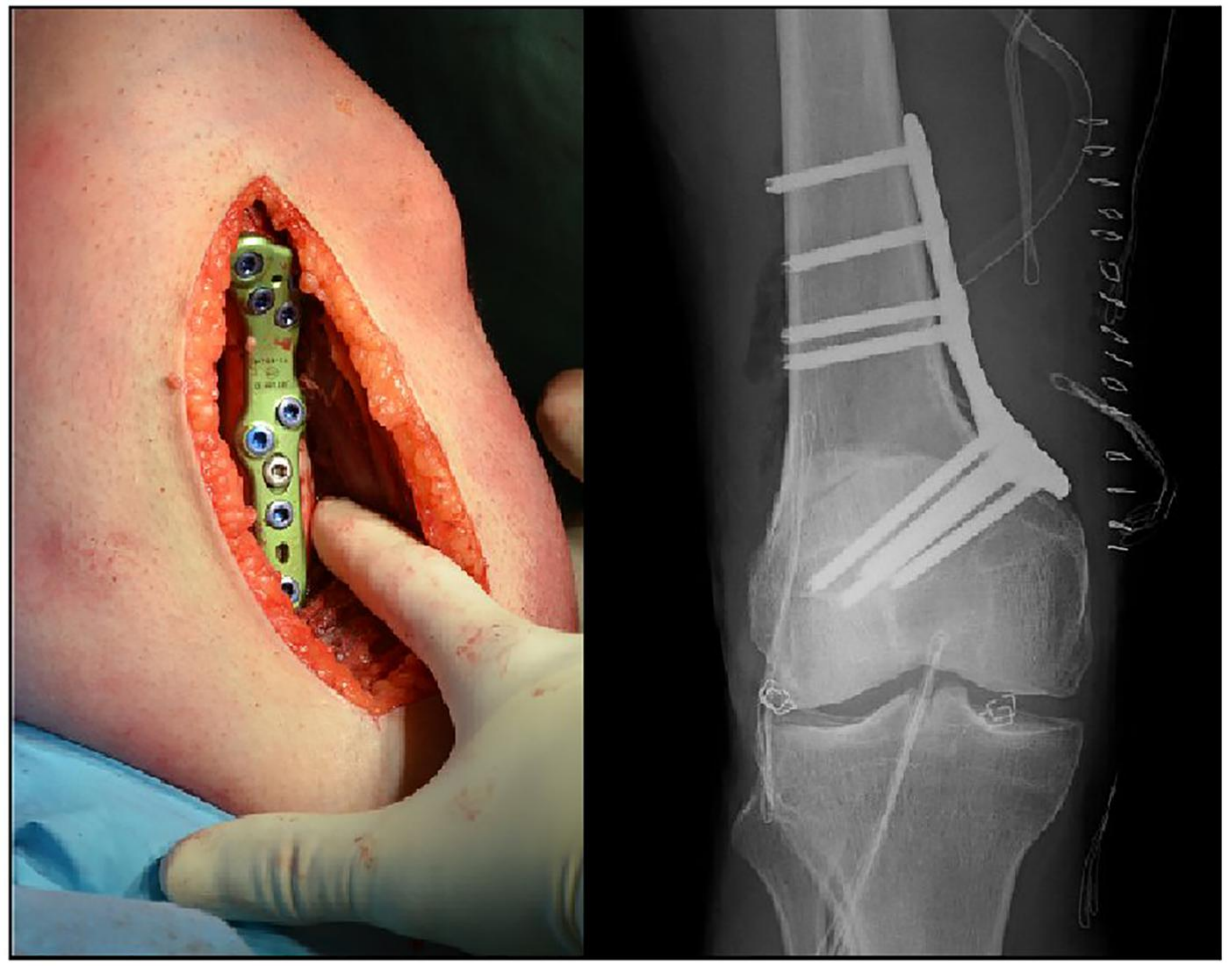
Osteotomy stabilized with a plate and angular stability screws.

After completing the osteotomy, the cutting guide, pins, and Kirschner wires are removed, and the plate is applied, which is stabilized with angular stability screws placed in a precise sequence to compress the osteotomy line (Figure 7). Before finishing the layered suture, it is advisable to place a subfascial drain. After surgery, a hinged knee brace is utilized, permitting either partial or full weight-bearing, depending on the bone quality and the stability obtained from the fixation device.

## Results

No statistically significant differences were observed between the two groups regarding demographic data, clinical characteristics, or preoperative radiographic parameters (Table 2). Likewise, no significant differences were found in the planned radiographic measurements of the mechanical femorotibial angle (mTFA) and mechanical lateral distal femoral angle (mLDFA) between the PSI and conventional surgery groups.

**Table 2:** Comparison groups: preoperative demographic, clinical, and radiographic characteristics.

Characteristic	MCWDFO-CONV	MCWDFO-PSI	*p* value
Number	10	16	/
Age (range)	55.6 (50–67)	53.4 (27–58)	0.71
Gender (M:F)	4:6	5:11	>0.05
BMI	24.7 (21.8–29.6)	23.8 (20.5–25.0)	0.19
ROM-Fl° (range)	128.6 (120.0–140.0)	130.4 (120.0–140.0)	0.53
pre-IKDC° (range)	58.6 (48.0–62.0)	61.1 (50.0–70.0)	0.23
K-L (I:II:III)	1:6:3	3:10:3	/
pre-*mFTA*° (range)	173.3 (166–178)	173.5 (167–176)	0.87
pre-*mLDFA*° (range)	82 (81–85)	83.4 (81–85)	0.29

BMI, body mass index; ROM-Fl, range of motion in flexion; pre-IKDC, preoperative IKDC score; K-L, Kellgren-Lawrence grade of osteoarthritis; pre-mFTA, preoperative mechanical femoro-tibial angle; pre-mLDFA, preoperative mechanical lateral distal femoral angle.

However, a significant difference was noted in postoperative mLDFA values (*p* < 0.01), with a mean of 85.7° (SD ± 1.39) in the MCW-DFO-CONV group and 88.2° (SD ± 1.21) in the MCW-DFO-PSI group. No significant difference was observed in postoperative mTFA, with mean values of 179.0° (SD ± 1.96) in the conventional group and 179.8° (SD ± 0.52) in the PSI group. Notably, the higher standard deviation in the conventional group indicates greater variability in alignment correction (Table 3).

**Table 3:** Radiographic results in the two comparison groups.

Characteristic	MCWDFO-CONV	MCWDFO-PSI	*p* value
plan-*mFTA*° (range)	179.4 (178.0–181.0)	179.8 (179.0–180.0)	0.32
plan-*mLDFA*° (range)	87.3 (85.0–90.0)	88.3 (85.0–90.5)	0.17
post-*mFTA*° (range)	179.0 (177.0–182.0)	179.8 (179.2–180.7)	0.41
post-*mLDFA*° (range)	85.7 (84.1–88.7)	88.2 (84.8–89.6)	<0.01
ε-*mFTA*° (±SD)	2.16 (±0.41)	0.46 (±0.29)	<0.01
ε-*mLDFA*° (±SD)	2.48 (±1.35)	0.66 (±0.51)	<0.01

plan-mFTA, planned mechanical femoro-tibial angle; plan-mLDFA, planned mechanical lateral distal femoral angle; post-mFTA, postoperative mechanical femoro-tibial angle; post-mLDFA, postoperative mechanical lateral distal femoral angle; ε-mFTA, absolute error between pre- and post-op mFTA; ε-mLDFA, absolute error between pre- and post-op mLDFA.

A significant difference was also observed in the comparison of absolute errors. The conventional group showed a mean ε-mFTA of 2.16° (SD ± 0.41) and ε-mLDFA of 2.48° (SD ± 1.35), while the PSI group demonstrated lower mean values of 0.46° (SD ± 0.29) and 0.66° (SD ± 0.51) respectively.

Regarding the secondary endpoints, the statistical analysis did not find a significant difference between the conventional group in terms of pre- and post-operative ROM or IKCD scores (*p*-values of 0.53 and 0.43, respectively). Fluoroscopy and surgical times were significantly lower in the PSI group, showing a mean of 14.4 s (SD ± 15.0) and 89.9 min (SD ± 5.0), respectively, compared to a mean of 32.3 s (SD ± 10.1) and 114.5 min (SD ± 20.6) in the conventional group (Table 4).

**Table 4:** Results in the two comparison groups.

Characteristic	MCWDFO-CONV	MCWDFO-PSI	*p* value
12 months ROM (range)	125.9 (120–140)	128.6 (120–140)	0.54
12 months IKDC (range)	83.7 (67–87)	84 (74–88)	0.43
Fluoroscopy, seconds (range)	32.3 (16–49)	14.4 (8–26)	<0.01
Surgical duration, minutes (range)	114.5 (80–145)	89.9 (70–125)	0.02
Intraoperative complications	3	0	/
Postoperative complications	0	0	/

## Discussion

The principal finding of the present study is that patient-specific instrumentation (PSI) significantly improves the accuracy and reproducibility of medial closing wedge distal femoral osteotomy (MCW-DFO) compared with conventional instrumentation. In addition to superior radiographic accuracy, the use of PSI was associated with reduced operative time and lower fluoroscopy exposure, without compromising short-term clinical outcomes or increasing complication rates. These findings suggest that PSI allows a more faithful intraoperative reproduction of preoperative planning in MCW-DFO, potentially enhancing surgical consistency and safety (21).

MCWDFO surgery for valgus knee correction serves as an effective method to halt or slow the degenerative changes in the lateral knee compartment, reduce pain, and enhance joint functionality (22, 23). The surgery aims to realign the load-bearing axis of the lower limb medially, relieving the lateral compartment from excessive strain while not significantly stressing the medial compartment. This requires thorough preoperative planning and a surgical method that guarantees accurate execution of the plan (24). This study primarily aims to showcase the simplicity of carrying out the preoperative plan for patients receiving surgery with PSI instrumentation. In this retrospective study, all patients undergoing MCWDFO underwent a thorough biometric evaluation using weight-bearing long-leg radiographs before surgery. The radiographic assessment included the mechanical femorotibial angle (pre-mFTA), which measures the overall degree of knee valgus, and the mechanical lateral distal femoral angle (pre-mLDFA), which indicates explicitly the effect of distal femoral metaphyseal deformity.

The surgical plan was created in TraumaCad®, where the surgeons simulated osteotomy and determined the amount of medial bone resection. This ensured that the postoperative load-bearing axis would pass through the centre of the knee. This addresses or corrects pre-arthritic valgus knees in cases of isolated lateral compartment chondropathy. Planned values for mFTA (plan-mFTA) and mLDFA (plan-mLDFA) were recorded and later analysed to assess surgical accuracy.

Postoperative radiographic evaluations enabled the calculation of the absolute error between the planned and actual alignment, providing a direct measure of the surgical correction's fidelity to the preoperative plan. In the conventional instrumentation group, the mean absolute error was 2.16° for mFTA and 2.48° for mLDFA. In contrast, the PSI group achieved significantly lower figures of 0.46° and 0.66°, respectively. These findings underscore the enhanced accuracy and reproducibility offered by patient-specific instrumentation in achieving desired corrections. Additionally, the lower standard deviations in the PSI group reflect not only improved mean accuracy but also a reduction in variability among patients, which highlights its potential advantages in ensuring surgical consistency. This is clinically important, as even slight deviations from the intended alignment can affect load distribution and long-term joint outcomes, especially in joint-preserving osteotomies procedures (25, 26). The noted improvement in reproducibility aligns with earlier studies on PSI-assisted osteotomies. It could be particularly beneficial for complicated deformities, where there is limited leeway for intraoperative errors.

Beyond numerical accuracy, the substantial reduction in alignment error observed in the PSI group further suggests that PSI not only supports precise osteotomy execution but may also enhance intraoperative decision-making by reducing reliance on fluoroscopic adjustments. This is important because intraoperative verification of the correction angle can be challenging, especially when soft tissues obscure anatomical landmarks. By providing a precontoured and positionally constrained guide, PSI appears to streamline this process, translating the digital plan more faithfully into the surgical field.

Despite the small sample size between the two groups, our findings indicate substantial differences in intraoperative ionizing radiation exposure and operative duration, thus minimizing risks for both patients and surgeons, especially infection and radiation exposure, respectively. There were no intraoperative complications in the PSI group. In contrast, the conventional group encountered lateral hinge fractures in two instances, suggesting a potential protective effect of the Kirschner wire on the hinge secured with the PSI guide.

No significant differences were observed in patient ROM or clinical satisfaction 12 months post-surgery, thus validating both techniques from this standpoint (27). In all cases treated, follow-up radiographs at the 12-month mark indicated healing at the osteotomy site. Although clinical outcomes were comparable between groups at 1 year, the enhanced radiographic accuracy in the PSI cohort may translate into long-term benefits, as previous studies have suggested that achieving a correction of target alignment is associated with improved survivorship of joint-preserving procedures (28). Longer-term follow-up will be necessary to determine whether the accuracy advantages of PSI ultimately influence osteotomy durability and reduce progression of lateral compartment degeneration.

### Relevance of accurate coronal alignment for meniscal and cartilage preservation

Accurate restoration of coronal limb alignment has important biomechanical and clinical implications, as even minor residual malalignment can significantly alter knee joint load distribution and contact stresses (29–31). Coronal plane malalignment, particularly valgus deformity, leads to a disproportionate increase in load transmission across the lateral compartment of the knee (29). The meniscus plays a key role in load sharing, shock absorption, and joint stability (32); however, a persistently altered mechanical environment exposes the meniscal tissue to excessive compressive and shear forces. Over time, such biomechanical alterations may result in meniscal degeneration and tearing (32, 33).

Once meniscal structure and function are compromised, its capacity to buffer and distribute joint loads is reduced, leading to a decrease in the effective contact area and a subsequent increase in focal cartilage stress. This process may accelerate articular cartilage degeneration and contribute to the progression of lateral compartment osteoarthritis (32, 33). From a clinical perspective, these mechanisms further highlight the importance of achieving accurate coronal alignment during distal femoral osteotomy. Therefore, the improved correction accuracy observed with PSI-assisted MCW-DFO may extend beyond short-term radiographic alignment, potentially contributing to the preservation of meniscal and cartilage integrity over time.

### Limitations

In addition to the considerations, the retrospective design of this study inherently exposes it to potential selection bias and limits the ability to establish causal relationships. The relatively small sample size reduces statistical power and may increase the risk of type II error, particularly for secondary outcomes. Furthermore, the absence of repeated radiographic measurements and formal inter- or intra-observer reliability assessment may affect the robustness of alignment accuracy evaluation. Variability in radiographic acquisition, including patient positioning and technician-dependent factors, was not formally standardized and may have introduced additional measurement error. These methodological constraints should be considered when interpreting the results. Future prospective studies with larger cohorts, standardized imaging protocols, and formal reliability analyses are necessary to confirm these findings and further validate the role of PSI in MCW-DFO.

## Conclusion

Medial closing wedge distal femoral osteotomy using NewClip Technics® patient-specific instrumentation provides high accuracy and reproducibility in achieving preoperative alignment targets. Compared to conventional instrumentation, PSI significantly reduces the absolute error in mFTA and mLDFA correction, shortens operative time, and decreases intraoperative fluoroscopy exposure. These findings support the reliability and potential clinical advantages of PSI-assisted MCW-DFO for correcting valgus knee deformities.

## Data Availability

The raw data supporting the conclusions of this article will be made available by the authors, without undue reservation.
